# SLC1A4 and Serine Homeostasis: Implications for Neurodevelopmental and Neurodegenerative Disorders

**DOI:** 10.3390/ijms26052104

**Published:** 2025-02-27

**Authors:** Dana Elazar, Natalie Alvarez, Sabrina Drobeck, Teresa M. Gunn

**Affiliations:** 1 Touro College of Osteopathic Medicine, Touro University, Great Falls, MT 59405, USA; delazar2@student.touro.edu (D.E.); nalvarez3@student.touro.edu (N.A.); sdrobeck@student.touro.edu (S.D.); 2 McLaughlin Research Institute, Great Falls, MT 59405, USA

**Keywords:** *solute carrier family 1 member 4* (*SLC1A4*) gene, SLC1A4, serine homeostasis, SPATCCM, neurodevelopment, neurodegeneration, Alzheimer’s disease

## Abstract

The *solute carrier family 1 member 4* (*SLC1A4*) gene encodes a neutral amino acid transporter, also referred to as alanine-serine-cysteine transporter 1, ASCT1, that helps maintain amino acid balance in the brain and periphery. In the brain, SLC1A4 plays an important role in transporting levo (L) and dopa (D) isomers of serine. L-serine is required for many cellular processes, including protein and sphingolipid synthesis, while D-serine is a co-agonist required for normal neurotransmission through *N*-methyl-D-aspartate receptors. Through its roles transporting L-serine across the blood–brain barrier and regulating synaptic D-serine levels, SLC1A4 helps establish and maintain brain health across the lifespan. This review examines the role of SLC1A4 in neurodevelopment and neurodegeneration and assesses the therapeutic potential of serine supplementation to treat neurodevelopmental symptoms associated with mutations in *SLC1A4*, as well as schizophrenia, depression, traumatic brain injury, and Alzheimer’s and Parkinson’s diseases.

## 1. Introduction

The *solute carrier family 1 member 4* gene (*SLC1A4*) encodes the neutral amino acid transporter, SLC1A4 (also called alanine-serine-cysteine transporter 1, ASCT1), which is involved in amino acid homeostasis [1,2]. It directs neutral amino acids, including serine, alanine, and cysteine, across cell membranes, which is necessary for maintaining neuronal function, synaptic transmission, and osmotic balance. In the brain, *SLC1A4* is expressed in astrocytes, with the highest levels found in the cerebral cortex, hippocampus, and cerebellum [3,4]. These brain regions are critical for cognition, memory formation, and motor control, making them fundamental to normal neurodevelopment. SLC1A4 activity ensures availability of L-serine, which is required for the production of the *N*-methyl-D-aspartate (NMDA) glutamate receptor co-agonists, glycine and D-serine, and it further modulates NMDA receptor activity by regulating synaptic levels of D-serine [3,5,6]. SLC1A4 is also expressed in peripheral tissues, such as the liver, kidney, skeletal muscle, and placenta, and has recently been identified in brain endothelial cells and shown to play a critical role in transport of L-serine across the blood–brain barrier (BBB) in mice [7,8]. Mutations in *SLC1A4* can disrupt transporter activity, resulting in altered amino acid transport for serine, alanine, cysteine, and threonine, deficiency of BBB-mediated L-serine transport and brain L-serine levels, and modified neurotransmitter precursor availability and neural signaling [3,9,10]. Reduced SLC1A4 levels or activity is associated with neurodegenerative disorders and conditions characterized by metabolic stress, suggesting a possible role in the pathogenesis of several neurologic and neurodegenerative disorders [3]. This review focuses on the importance of serine metabolism in the brain and the emerging insights into the role of SLC1A4 in maintaining serine homeostasis in neurodevelopment and brain aging.

## 2. SLC1A4 and Serine Metabolism

**Figure 1:**
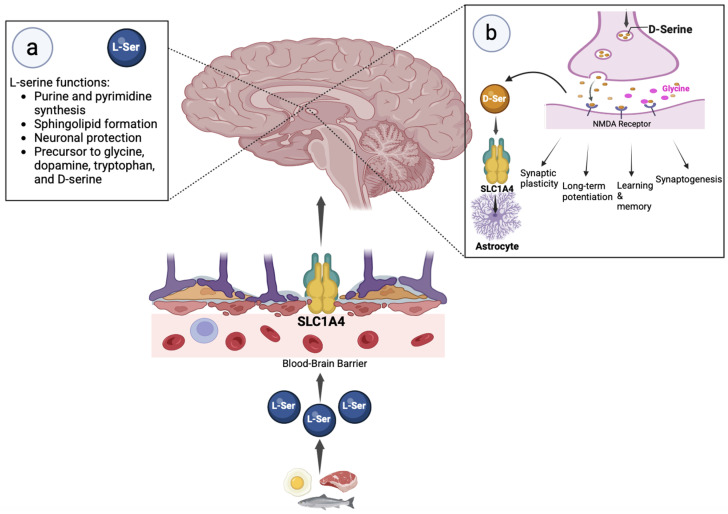
Serine transport into and function in the brain. L-serine is ingested via dietary sources such as meat, eggs, fish, and legumes and is transported into the brain across the BBB via SLC1A4 and other transporters. Panel (**a**) illustrates the key functions of L-serine (L-ser) in the brain and underscore its importance in both cellular metabolism and neurological health. L-serine is converted to D-serine (D-ser) by the enzyme serine racemase in neurons. Panel (**b**) illustrates D-serine secretion into the synaptic cleft where it functions as a co-agonist for NMDA receptors (right), serving as a critical component in synaptic plasticity, long-term potentiation, learning and memory, and synaptogenesis. SLC1A4, expressed on astrocytes (left), is the major uptake system to remove excess D-ser from the synaptic cleft. Not shown: astrocytic SLC1A4 can also heteroexchange L-ser for D-ser, providing neurons with the L-ser needed for D-ser synthesis.

Serine exists in two enantiomers: L-serine and D-serine. Both play important roles in a variety of metabolic processes. L-serine can be synthesized de novo, but most is derived from diet. It is found in a number of natural compounds, such as dairy products, legumes, fish, meat, and eggs (Figure 1). L-serine plays key roles in purine and pyrimidine synthesis and sphingolipid formation, and serves as a precursor to dopamine, glycine, and tryptophan in addition to D-serine (Figure 1a) [11]. L-serine has demonstrated neuroprotective properties by reducing reactive oxygen species, suppressing microglial activation, and decreasing the production of inflammatory mediators such as TNF-*α*, IL-6, and IL-1 in the brain [12,13,14,15]. Conversely, D-serine must be synthesized in the body. Accumulating evidence suggests that in the brain, D-serine is predominantly synthesized in neurons, supported by the observation that mice homozygous for neuron-specific knock-out of serine racemase (the enzyme that isomerizes L-serine into D-serine) have endogenous D-serine levels < 15% of that of controls [16,17,18]. Neuronal synthesis of D-serine utilizes L-serine produced in astrocytes by the enzyme 3-phosphoglycerate dehydrogenase (PHGDH), suggesting a serine shuttle mechanism between astrocytes and neurons [19,20,21,22,23,24,25]. Recent work also supports a role for SLC1A4 in the transport of L-serine across the BBB (Figure 1) [8].

D-serine functions as a neuromodulator in the central nervous system through its role as a co-agonist of NMDA receptors (Figure 1b) [26,27]. The activation of tetrameric NMDA receptors is triggered by the binding of synaptically released L-glutamate to GluN2 (or GluN3) subunits. Receptor activation also requires the binding of glycine (extrasynaptically) or D-serine (at synaptic NMDA receptors) to co-agonist sites on GluN1 subunits [27,28,29]. While the ambient extracellular concentration and relative contribution of each co-agonist appears to depend on age and brain region, evidence suggests that D-serine plays an equal if not greater role in the control of synaptic NMDA receptor signaling in adult cortices [26,27,30,31,32]. Membrane transport is likely to be critical in determining the ambient concentrations of NMDA receptor co-agonists. SLC1A4 is expressed by astrocytes and plays a major role in D-serine reuptake in the brain (Figure 1b) [5].

## 3. SLC1A4 in Neurodevelopment

### SPATCCM

An important role for SLC1A4 in human brain development was revealed when recessive mutations in *SLC1A4* were identified in children with spastic tetraplegia, thin corpus callosum, and progressive microcephaly (SPATCCM) [9,33,34,35,36,37,38,39,40]. More recently, a dominant mutation was described that duplicates three amino acids (L86_M88dup), resulting in a dominant-negative N-glycosylation defect that reduces SLC1A4 plasma membrane localization and L-serine transport [41]. Primary clinical manifestations in these patients include intellectual disability, microcephaly, speech delay, hypotonia (which can range from mildly clumsy to wheelchair-bound), and movement disorders (Table 1). Patients may also show growth retardation and failure to thrive, behavioral abnormalities, ataxia, metabolic disturbances, and seizures. The variable severity of clinical phenotypes may reflect genetic modifiers, environmental factors, and/or epigenetic effects. The majority of SPATCCM patients have heterozygous consanguineous parents and most common mutations are point mutations that alter single amino acids critical for SLC1A4 function, including p.E256K and p.R457W, but loss-of-function mutations have also been identified, including a nonsense mutation (c.573T > G, p.Y191*) that most likely results in nonsense mediated decay and no production of SLC1A4 protein. Interestingly, p.E256K mutant SLC1A4 shows increased affinity for D-serine but a lower maximum velocity, consistent with saturation at a lower concentration [10]. To date, no mutations have been described that result in upregulation of *SLC1A4*.

Mouse models provide an opportunity to investigate the effects of specific genetic changes associated with human diseases and provide a better understanding of gene function. Two *Slc1a4* mouse models have been developed that, interestingly, show divergent phenotypes from each other and from human patients with SPATCCM (Table 1). *Slc1a4* knock-out mice were generated by the Knockout Mouse Project (KOMP) on a C57BL/6N background [42], while two knock-in models have been described that carry the p.E256K mutation described in several human SPATCCM patients: one generated using traditional gene-targeting approaches in 129B6 hybrid ES cells and backcrossed onto the C57BL/6J background for five generations [8] and another generated using CRISPR-Cas9 gene editing on a C57BL/6J background [10]. The knock-out model had lower levels of brain D-serine but higher levels of L-alanine, L-threonine, and glycine [3]. They also showed significantly decreased D-serine and L-serine transport across astrocytes, while the knock-in models demonstrated higher uptake of D-serine in homozygotes [8,10]. *Slc1a4* knock-out and p.E256K knock-in mice showed reduced striatal and hippocampal volumes, enlarged ventricles, and had reduced brain weight, but only p.E256K homozygotes displayed a thin corpus callosum (Table 1). *Slc1a4* knock-out mice displayed mild sensorimotor, behavioral, and learning deficits, showing poorer initial performance on the rotarod and in the Morris water maze but similar performance to controls at later timepoints, suggesting they may learn more slowly but retained information once it was learned. The two p.E256K models showed different behavioral phenotypes, perhaps reflecting differences in genetic background: at 5 months-of-age, the CRISPR-Cas9 generated mice showed no differences relative to control mice on the Barnes maze or rotarod but had reduced exploratory behavior in the novel object test, while 90 day-old homozygotes for the allele generated using a traditional gene targeting approach did display impaired performance on rotarod. Although patients with SPATCCM present with reduced comprehension and verbal skills, which may be related to impaired NMDAR signaling due to altered D-serine homeostasis, none of the *Slc1a4* mouse models reported to date show defects in long-term potentiation (LTP) in the hippocampus. Another difference in the clinical presentation of SPAATCC patients and the mouse models is seizure susceptibility (Table 1), although the fact that not all human patients have seizures suggest there may be modifier genes and/or environmental factors that influence the penetrance of this phenotype.

A key finding from mouse model studies is that SLC1A4 is expressed in brain endothelial cells and plays a significant role in transporting L-serine across the BBB, especially developmentally [8]. Mice homozygous for the *Slc1a4* knock-out or the p.E256K mutation demonstrated a reduced influx of serine through the BBB, and wildtype mice treated with a SLC1A4-specific inhibitor that can act on vascular cells but does not cross the BBB displayed a significant decrease in brain L-serine, whereas knock-out mice treated with the drug showed no change. Furthermore, conditional deletion of *Slc1a4* in *Tie2*-expressing endothelial cells recapitulated many of the *Slc1a4* mutant phenotypes. Consistent with a deficiency of BBB-mediated L-serine transport in the mutant mice, doubling oral intake of L-serine of mothers, starting two days before pups were born through to postnatal day 11 resulted in improved brain weight and motor performance in p.E256K homozygous mutant offspring. Brain L-serine levels were increased but, somewhat surprisingly, there was no effect on brain D-serine levels. Taken together, these data suggest that the clinical phenotypes of SPATCCM patients may also be predominantly due to defective transport of L-serine across the BBB and that serine supplementation may be beneficial, although it is unclear at what developmental stage supplementation would need to be initiated to have a significant impact.

The differences in the clinical presentation of human SPATCCM patients and mice homozygous for knock-out or knock-in mutations in *Slc1a4*, summarized in Table 1, suggest there may be species-specific compensatory mechanisms, perhaps reflecting differences in serine transport and metabolism. In mice, alternative pathways or regulatory networks might buffer the effects of *Slc1a4* mutations, maintaining partial D-serine regulation and mitigating more severe phenotypes observed in human SPATCCM patients. These mechanisms could include differences in the expression patterns of other amino acid transporters, enzyme activity, or alternative metabolic pathways that compensate for disrupted SLC1A4 function. Genetic or environmental factors may also modify the penetrance of SPATCCM phenotypes. Another consideration is species-specific differences in NMDA receptor subunit composition and D-serine regulation, particularly between rodents and humans, which influence behaviors, synaptic function, and responses to neurological disorders [43]. Further investigation into the cause of the species differences in phenotype associated with impaired SLC1A4 function could reveal novel therapeutic approaches. The limitations these differences impose must also be taken into consideration when using the mouse models to test treatment strategies for SPATCCM.

## 4. Neuropsychiatric Disorders

### 4.1. Schizophrenia

Schizophrenia is a mental disorder distinguished by symptoms of disordered thoughts, delusions, and hallucinations. It has been proposed that glutamatergic dysfunction underlies its pathogenesis [44,45,46]. This hypothesis is supported by the significant association of schizophrenia with glutamate receptor genes and genes related to glutamatergic neurotransmission [47,48], as well as the observation that exposing healthy individuals to drugs like phencyclidine (PCP) or ketamine, which block NMDA receptors, produces behaviors resembling the symptoms and cognitive deficits of schizophrenia, while treating individuals with schizophrenia with these drugs exacerbates their symptoms [49,50]. Deng and colleagues [44] assessed whether genes involved in glutamatergic transmission, including *SLC1A4,* were associated with schizophrenia using 100 Japanese case–control pairs. Although they found nominally significant associations between two single nucleotide polymorphisms (SNPs) in *SLC1A4,* the association did not survive controlling for the false discovery rate at level 0.05 and they concluded that this gene is not a major susceptibility locus in the Japanese population. Another study assessing a German population also failed to detect an association between *SLC1A4* and schizophrenia [51], while earlier studies that mapped schizophrenia susceptibility to the same chromosomal region as *SLC1A4* had failed to identify any variants within SLC1A4 that segregated with schizophrenia [47,48]. Studies have reported low serum D-serum levels in people with schizophrenia, however (see Cho et al., 2016 for a meta-analysis of 20 studies [52]), and although studies have failed to identify a genetic association between SLC1A4 and schizophrenia, *SLC1A4* was identified as a gene with dysregulated expression in the cortical brains of individuals affected with schizophrenia [53], suggesting that it may be involved in disease pathogenesis but acts downstream of causative genes.

### 4.2. Depression

Major depressive disorder (MDD; depression) is a common chronic disease with mental, emotional, and physical health effects [54]. Symptoms include sadness, low self-esteem, sleep disturbance, tiredness, attention deficit, and reduced motivation or ability to experience pleasure, and patients frequently show intention to self-harm or commit suicide. Altered serine metabolism has been implicated in the pathophysiology of MDD through its role in modulating NMDA receptor-mediated neurotransmission, and D-serine and the NMDA antagonist ketamine have been shown to have anti-depressive effects in rodent models of depression [55,56,57] and clinical trials with human patients [58,59,60,61,62,63,64,65]. Furthermore, an integrative gene set-based GWAS meta-analysis of case–control studies of MDD revealed statistical enrichment for variants in genes involved in glutamatergic synaptic neurotransmission, including *SLC1A4* [66], and reduced SLC1A4 expression has been reported in the anterior cingulate cortex and hippocampus of patients with MDD [67]. Altered SLC1A4 levels or activity would lead to impaired inhibitory control of glutamatergic neurotransmission and result in hyperexcitability of cortical neurons, a phenomenon linked to disrupted affective processing and observed in MDD (reviewed in [68]). While studies report conflicting data on whether L- and D-serine levels are elevated in the serum or CSF of MDD patients compared to healthy controls, CSF D-serine concentration correlated with MDD severity (based on the Hamilton Depression Rating Scale) and baseline D-serine plasma concentration may predict ketamine responsiveness [69,70,71].

## 5. SLC1A4 and Serine Metabolism in Aging and Neurodegeneration

### 5.1. Normal Aging

Aging is characterized by a decline in various brain functions, including cognitive performance and neuronal plasticity [72,73]. Proper amino acid transport is essential for maintaining neuronal health and supporting neurotransmitter systems, including NMDA receptor signaling. During normal aging, there is reduced expression of serine racemase, leading to lower levels of D-serine and decreased NMDA receptor activity, and this down-regulation is believed to impair synaptic plasticity, resulting in deficits in learning and memory [43,73]. As discussed earlier in this review, SLC1A4 plays a crucial role in transporting neutral amino acids, which are critical for maintaining neurotransmitter balance and neuronal health. D-serine is a co-agonist of NMDA receptors, which are essential for synaptic plasticity and memory formation. Dysregulation of NMDA receptor activity has been implicated in age-related cognitive decline as well as AD [74]. Activation of NMDA receptors depends on the co-agonist site being occupied, consistent with the idea that endogenous D-serine levels significantly influence neuronal plasticity, both during development and in the mature brain [5]. Since SLC1A4-mediated reuptake of D-serine into astrocytes plays an important role in regulating synaptic D-serine levels, SLC1A4 levels and activity affect age-related cognitive decline and AD. The observations that plasma D-serine levels decrease with age and D-serine supplementation can reverse age-related cognitive decline in rats has led to the hypothesis that cognitive decline in older individuals may be associated with a deficiency of this critical co-agonist [75]. As discussed in the next sections, several studies have explored the relationship between serine levels and neurodegenerative diseases as well as the possible use of serine supplementation in disease management.

### 5.2. Alzheimer’s Disease

Alzheimer’s disease (AD) is the most common cause of late-onset dementia, affecting nearly 7 million Americans aged 65 and older [76]. While reduced D-serine levels have been linked to cognitive decline associated with normal aging, studies report conflicting results on D-serine levels in the brains of individuals with AD and AD mouse models. One study reported reduced levels of PHGDH in hippocampal astrocytes in AD brains as well as in a 3xTg transgenic mouse model that expresses three mutations associated with familial AD [77]. Consistent with PHGDH’s role in catalyzing the first rate-limiting step in converting glucose to L-serine, both L- and D-serine levels were reduced in the brains of 3xTg mice; however, levels were not assessed in AD brain tissue. In the mice, recordings of the NMDA receptor component of field excitatory post-synaptic potentials in acute hippocampal slices indicated a lower level of co-agonist binding site occupancy in 3xTg mice relative to controls. Conversely, elevated levels of D-serine have been detected in serum and CSF from AD patients compared to healthy controls [78,79,80]. Furthermore, SLC1A4 and D-serine levels were increased in the brains of two other mouse AD models. In 5xFAD transgenic mice, which overexpress mutant human amyloid precursor protein harboring five familial AD (FAD) mutations as well as human presenilin-1 harboring two FAD mutations, SLC1A4 levels were twice as high as in wildtype controls, as were cortical levels of serine [81]. Similarly, the brains of 12-month-old knock-in mice expressing human amyloid precursor protein harboring four FAD mutations also showed elevated levels of SLC1A4 and D-serine [82]. Taken together, these observations underscore the importance of astrocytes in serine metabolism and suggest that altered SLC1A4 activity could contribute to AD by impacting the availability of D-serine on NMDA receptors. It is unclear what triggers the increase in SLC1A4 observed in the brains of mouse AD models, or whether a similar increase occurs in human AD brains. The conflicting observations on D-serine levels in biofluids and PHGDH levels in AD brains, and in the brains of different mouse AD models, indicate that further studies of human AD brain tissue are needed. The dichotomy if D-serine levels decrease in the brain with normal aging but increase in AD brains could be explained by the fact that NMDA receptor hypo- and hyperactivity can both lead to synaptic dysfunction, neurotoxicity, and cognitive dysfunction [72,83].

### 5.3. Parkinson’s Disease

Parkinson’s disease (PD) is a progressive neurological condition characterized by motor symptoms, constipation, depression, memory loss, and difficulty swallowing and speaking [84]. It is associated with the degeneration of neurons in the brain that produce dopamine and norepinephrine. Altered NMDA receptor neurotransmission has been observed in the striatum of PD patients and is thought to contribute to the onset and progression of PD symptoms [85,86,87]. Disrupted serine homeostasis is further implicated in PD pathogenesis by several studies. One reported that, although there were no differences in total serum levels of D- or L-serine between PD patients and healthy controls, changes in serum D-serine levels correlated with age and age-of-onset in PD patients [14]. Another study demonstrated a significant reduction in SLC1A4 and a significant increase in D-serine levels in post-mortem caudate putamen samples from PD patients compared to healthy controls [88] and that D-serine was significantly increased in CSF of living PD patients. However, similar to the contradictory results reported for D-serine levels in AD, other studies have reported lower D-serine in the substantia nigra and cerebrospinal fluid of untreated PD patients [89]. These studies suggest a link between SLC1A4, serine homeostasis, and PD, but the underlying mechanisms remain unclear. Further evidence that serine levels are important in PD pathogenesis comes from studies examining serine supplementation as a treatment for PD, which will be discussed below.

### 5.4. Traumatic Brain Injury

Traumatic brain injury (TBI) is a form of acquired brain injury resulting from sudden trauma to the brain that disrupts homeostasis, with severity ranging from mild concussion to severe trauma with acute or chronic cognitive dysfunction [90]. Brain trauma is associated with neuroinflammation and a widespread gliotic response, involving alterations in astrocyte and microglial morphology and the proteins they express [91,92]. One of these changes includes an increase in D-serine production, associated with a switch in its production from neurons to astrocytes, which is thought to contribute to synaptic damage and dysfunction [93]. One study demonstrated that SLC1A4 was up-regulated in the cortex of mice following TBI induced by controlled cortical impact, and treatment with a noncompetitive substrate inhibitor with high selectivity and potency for SLC1A4 or with genetic ablation of *Slc1a4* in astrocytes or microglia was protective [94]. That study also demonstrated up-regulation of SLC1A4 mRNA and protein in peri-lesional tissues resected from human TBI patients relative to cortical control specimens, further linking SLC1A4 and D-serine to TBI pathology.

## 6. Serine Supplementation

Given the connections between brain serine levels and cognition, as well as with multiple neurodevelopmental and neurodegenerative diseases, there is increasing interest in the therapeutic potential of serine supplementation. There are competing trains of thought regarding the safety and benefits of L-serine or D-serine supplements. L-serine is currently FDA approved, while D-serine is not. Moreover, L-serine is a neurotrophic factor, and its neuroprotective properties are thought to be superior to those of D-serine, the latter having more potential for possible side effects related to NMDA receptor-mediated excitotoxicity. The likelihood of encountering this undesirable side effect increases in patients with mutations that affect D-serine transport, whereas excess L-serine does not appear to be associated with adverse effects [15]. L-serine also plays important roles in the proliferation and survival of neural stem cells as well as microglial activation and cytokine production, processes central to protection against and recovery from nervous system injury and disease (reviewed in [12]). On the other hand, L-serine must be converted into D-serine to achieve the desired therapeutic outcome, and the pathway responsible for this process may be altered in some patients, which could weaken the efficacy of L-serine supplementation [15]. Below, we summarize the therapeutic potential of serine supplementation for the treatment or management of neurodevelopmental and neurodegenerative disorders associated with perturbed SLC1A4 function and/or serine homeostasis.

Serine supplementation has been suggested as a treatment for SPATCCM patients, with the caveat that it is unclear whether treatment started after diagnosis would be too late to have a beneficial effect [33,35,37,40]. Mouse models have provided some insight into this issue. Pregnant dams carrying *Slc1a4*-p.E256K homozygous pups were supplemented with L-serine in their drinking water starting 2–3 days before pups were born, and brain weight, ultrasonic vocalizations, and motor performance of the offspring were assessed on postnatal day 11 [8]. All three parameters were normalized and associated with increased levels of brain L-serine, but not D-serine. The latter observation is surprising, given that D-serine is synthesized from L-serine, but suggests that the phenotypes assessed specifically reflect a deficiency in brain L-serine. Further studies are needed to determine whether there are any beneficial effects of treatment at later stages, corresponding to the mouse equivalent of the age at which human SPATCCM patients are typically diagnosed, and whether individuals with null mutations (i.e., that lack SLC1A4 expression altogether) benefit from dietary serine supplementation.

Augmentation of antipsychotic treatments with NMDA receptor modulators, including D-serine, improved multiple schizophrenia symptoms, particularly negative ones, with satisfactory side effects and safety profile (see Goh et al., 2021 for meta-analysis of 40 trials involving 4937 patients with schizophrenia [95]). More recently, a double-blind, placebo-controlled study demonstrated a beneficial effect of D-serine supplementation in combination with auditory cognitive remediation treatment, with a dose-dependent improvement in plasticity [96]. These findings underscore the critical role of NMDA receptor function in schizophrenia and highlight D-serine supplementation as a promising therapeutic avenue to enhance neural plasticity and potentially mitigate disease symptoms. In fact, D-serine supplementation may be beneficial for a broad range of psychiatric disorders as many are associated with altered signaling through NMDA receptors, including MDD [97,98]. The role of D-serine in depression is supported by studies in mice. Serine racemase knock-out mice have dramatically reduced D-serine levels and exhibited elevated levels of anxiety and reduced cognitive function [16]. Transgenic over-expression of serine racemase in astrocytes and acute or long-term administration of D-serine resulted in elevated brain D-serine levels and was associated with behavioral phenotypes similar to those observed in mice treated with anti-depressant drugs [55,56].

Oral serine supplementation has also been suggested as a “ready to use therapy for AD” [77]. In 3xTg AD mice, reduced levels of astrocytic PHGDH and hippocampal L-serine and D-serine correlated with impaired synaptic plasticity and deficits in learning and memory. Oral supplementation with D-serine in drinking water at levels known to increase D-serine in the brain rescued the cognitive deficits of 6- to 7-month-old female 3xTg-AD mice [77]. This study also showed that dietary L-serine supplementation restored D-serine levels, improved NMDA receptor activity, and protected against synaptic and cognitive deficits. These observations suggest that L-serine supplementation is as effective as D-serine in counteracting the metabolic deficiencies caused by AD and in supporting essential neuronal functions, an important finding since L-serine supplements are FDA approved and safe. However, further clinical research is needed to confirm these results in human patients and evaluate the long-term safety and efficacy of L-serine supplementation in treating AD. Furthermore, as described earlier in this review, there is conflicting data on D-serine and PDGDH levels in AD patients. Given the discrepancies that have been reported, it is not surprising that other studies have cautioned against the use of serine as a therapy for AD [99]. Moving forward, further research is needed to better understand the role of PHGDH and serine metabolism in AD progression and to inform therapeutic strategies.

PD is currently treated with levodopa (L-DOPA) supplementation, but chronic use is associated with a multitude of side effects, including deficits in motor function as well as L-DOPA-induced dyskinesia [84], and its effectiveness wanes over time. Thus, new or combinatorial therapeutic approaches are still much needed. A double-blind, placebo-controlled study conducted over a 6-week period suggested a positive correlation between a 30 mg/kg per day dose of D-serine and the relief of motor and extrapyramidal symptoms in thirteen PD patients [100]. Further studies are needed to replicate the results in a larger cohort, determine optimal dose, and test whether L-serine replicates the effects, but these preliminary findings hint at potential benefits of serine supplementation to PD patients.

## 7. Conclusions

While the importance of D-serine in neurotransmission and synaptic plasticity is well established, the neuroprotective and neurorestorative effects of D-serine supplementation to treat neurodevelopmental and neurodegenerative disorders remain somewhat controversial, perhaps reflecting the fact that too much or too little NMDA receptor signaling can be detrimental to brain health. Given the role of astrocytic SLC1A4 in mediating the exchange of intracellular L-serine for extracellular D-serine, this transporter may represent a key therapeutic target for maintaining serine homeostasis in the brain. While L- or D-serine supplementation may be beneficial in cases where D-serine levels are reduced, it may also be possible to reduce SLC1A4 activity using selective, high-affinity inhibitors such as the alkoxy hydroxy-pyrrolidine carboxylic acid compounds described by Lyda et al. [101]. These inhibitors pharmacologically modulate transporter function and, if they cross the BBB, could be used to treat AD, TBI, and other brain disorders associated with NMDA-mediated excitotoxicity due to increased SLC1A4-mediated D-serine efflux from inflammatory astrocytes. Furthermore, discovering pathways that regulate SLC1A4 levels and activity may reveal ways to up-regulate it to treat diseases associated with elevated extra-synaptic D-serine, although further studies are needed to assess potential side-effects.

## Figures and Tables

**Table 1:** Comparison of clinical phenotypes of SPATCC patients and *Slc1a4* mouse models.

Symptom	SPATCCM (Various *SLC1A4* Mutations)	*Slc1a4^tm1e(KOMP)Wtsi^* (Knock-out, C57BL/6N)	*Slc1a4^em2Tmg^* (p.E256K, C57BL/6J)	*Slc1a4^tmColumbia^* (p.E256K, B6;129B6)	*Slc1a4^fl/fl^; Tg.Tie2-cre* (Endothelial Knock-out)	*Slc1a4^p.E256K^* + Maternal Serine Supplementation
Intellectual Disability	+	+/−	−	+	+	Improved
Microcephaly	+	+	+	+	+	Improved
Thin Corpus Callosum	+	N/A	+	+	N/A	N/A
Seizures	+/−	N/A	−	N/A	N/A	N/A
Impaired Motor Coordination	+	+	−	+	+	Improved
Growth Retardation	+	−	−	−	−	−
Ataxia	+	−	−	N/A	N/A	N/A
Metabolic Disturbances	+	+	N/A	+	+	+/−
Altered Serine Uptake	+	+	+	+	+	Normalized

Legend: present (+), absent (−), variable (+/−), not explicitly reported (N/A).

## Data Availability

Not applicable.

## Abbreviations

The following abbreviations are used in this manuscript:

AD	Alzheimer’s disease
ASCT1	Alanine-serine-cysteine transporter 1
BBB	Blood–Brain Barrier
CSF	Cerebrospinal fluid
D-ser	Dextro (D)-serine
FAD	Familial Alzheimer’s Disease
GluN2	Glutamate ionotrophic receptor NMDA type subunit 2
GluN3	Glutamate ionotrophic receptor NMDA type subunit 3
KOMP	Knockout Mouse Project
L-DOPA	Levodopa
L-ser	Levo (L)-serine
LTP	Long-term potentiation
MDD	Major Depressive Disorder
NMDA	*N*-methyl-D-aspartate
PD	Parkinson’s disease
PCP	Phencyclidine
PHGDH	Phosphoglycerate dehydrogenase
*SLC1A4*	*Solute carrier family 1 member 4 gene*
SLC1A4	Solute carrier family 1 member 4 protein
SNP	Single nucleotide polymorphism
SPATCCM	Spastic tetraplegia, thin corpus callosum and progressive microcephaly
TBI	Traumatic Brain Injury
